# 1211. Assessing the Applicability of Enhanced Barrier Precautions among Adult Hospitalized Patients

**DOI:** 10.1093/ofid/ofac492.1044

**Published:** 2022-12-15

**Authors:** Jinal Makhija, Michael Y Lin

**Affiliations:** Rush University Medical Center, Orland Park, Illinois; Rush University Medical Center, Orland Park, Illinois

## Abstract

**Background:**

In nursing homes, the CDC has endorsed an interim approach for containment of multidrug-resistant organisms called Enhanced Barrier Precautions (EBP). With an EBP approach, residents with either indwelling medical devices or chronic wounds are considered at risk for multidrug-resistant organisms; therefore, healthcare personnel are to use gown and gloves for care activities that are considered high risk for organism transmission (e.g., dressing or bathing a patient), while lower risk activities are excluded. EBP guidance currently does not apply to acute care hospitals; we aimed to assess what proportion of hospitalized patients would qualify for an EBP prevention approach.

**Methods:**

We performed rolling single day point prevalence surveys for all adult inpatient units at Rush University Medical Center, Chicago, IL in March-April 2022. Using electronic chart review, we recorded patient unit location, multidrug-resistant organism colonization status, Contact Precautions status, presence of indwelling medical devices, and presence of wounds (pressure ulcer of stage ≥2, or open surgical wound). Patients with any indwelling device or wound qualified for EBP. We also assessed alternate definitions of EBP (device-only or wound-only). Prevalence differences were analyzed using the Chi-squared test.

**Results:**

We assessed 353 hospitalized patients (characteristics, Table 1). Among all patients, 18% (n = 65) were in Contact Precautions, primarily for the indication of multidrug-resistant organism or *C. difficile* control. Under an EBP approach, a higher proportion (52%, n = 184, *P* = .005) would qualify. Under alternate EBP definitions, 49% (n = 172) would qualify under a device-only criterion, and 9% (n = 32) would under a wound-only criterion. Comparing intensive care unit (ICU) vs non-ICU patients, Contact Precautions rates were similar, but EBP rates would be higher in ICU patients than non-ICU patients, driven by device use (Table 2).

**Figure:**
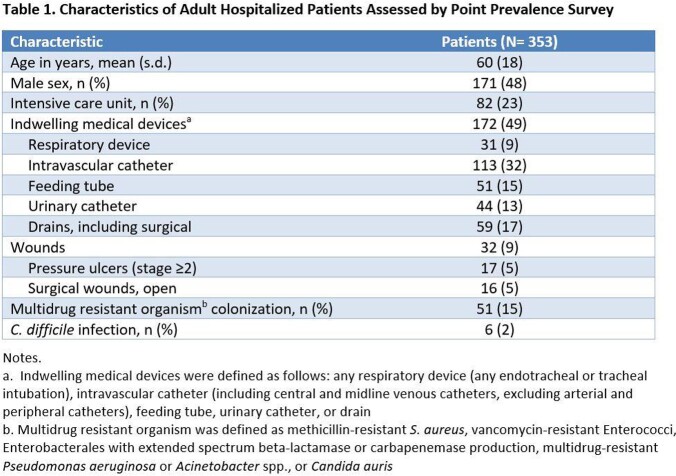

**Figure:**
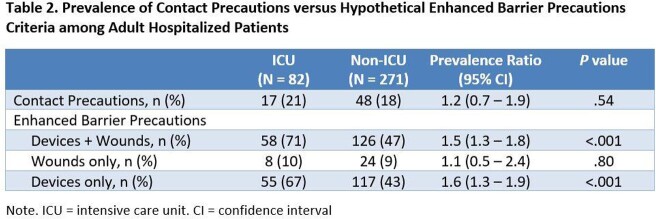

**Conclusion:**

An EBP infection control approach would impact a substantially larger proportion of hospitalized patients, compared to traditional indications for Contact Precautions. To improve feasibility of an EBP approach among hospitalized patients, further refinements to the qualifying criteria are likely needed.

**Disclosures:**

**All Authors**: No reported disclosures.

